# Extremely Preterm (23 Weeks) Vaginal Cephalic Delivery En Caul and Subsequent Postpartum Intraventricular Hemorrhage and Respiratory Distress: A Teaching Case

**DOI:** 10.1155/2018/5690125

**Published:** 2018-04-30

**Authors:** Rohail Malik, Adil Sarfraz, Raihan Faroqui, William Onyebeke, Jeffrey Wanerman

**Affiliations:** Department of Obstetrics & Gynecology, Southside Hospital, Northwell Health, 301 East Main Street, Bay Shore, NY 11706, USA

## Abstract

En caul deliveries are defined as a fetus that is delivered completely contained within an amniotic sac and are considered to be less common than 1 in 80,000 live births. Vaginal en caul births compared to abdominal or cesarean are the rarest subtype. Most en caul births are associated with prematurity and low gravida. The combination of prematurity, high gravida, vaginal en caul birth, and severe postpartum complications has not previously been described. We report a woman with gravida of 12 delivering vaginally a neonate female en caul at the extremely preterm gestational age of 23 weeks. The neonate subsequently decompensated, underwent respiratory distress, and was diagnosed with a bilateral intraventricular hemorrhage. Owing to deteriorating status, supportive care was removed and the infant was pronounced dead 5 days after delivery.

## 1. Introduction 

During pregnancy, the fetus is enclosed in an amniotic sac (also known as “amnion” or “caul”). In a normal delivery, the amniotic sac ruptures, and the fetus is delivered through the vaginal opening. A “caul” delivery occurs when a piece of the amniotic sac is still attached to the neonate at the time of delivery, usually attached to the head or torso [1, 2]. An “en caul” delivery, which is a subtype of caul delivery, occurs when the entire intact amnion is delivered with the neonate inside. Abdominal (or cesarean) en caul deliveries can be performed intentionally with surgical technique, whereas vaginal en caul deliveries occur spontaneously; as such, vaginal en caul deliveries are considered to be rarer though exact statistics are difficult to determine. According to some estimates, caul deliveries would be 1-2%, or roughly 1 in 80,000, of all vaginal deliveries if no membranes were artificially ruptured [2, 3].

Most en caul births are low gravida and premature. Prematurity is a significant risk factor for various postpartum complications. Previous case reports have poorly described pertinent maternal information beyond gestational age at birth (e.g., past medical history, past surgical history, family history, and demographic information) which could be used to formulate additional risk factors for en caul birth.

We will describe a case study of en caul delivery of a female infant at 23 weeks with a weight of 497 grams to a gravida 12 African-American female. Owing to intraventricular hemorrhage and respiratory distress, the infant died 5 days after delivery. We provide useful clinical parameters for management of en caul birth and postpartum complications with a review of the existing case literature.

## 2. Case Report

We report a case of a 38-year-old gravida 12 multiparous African-American woman with a history of six termination of pregnancies (TOP), five term uncomplicated normal spontaneous vaginal deliveries (NSVD), fibroids, gastroesophageal reflux disorder (GERD), and obesity presented at 23 weeks gestation by dates. The patient presented with contractions, 3 hours of worsening lower abdominal pain, and three episodes of vomiting. She denied vaginal bleeding, spontaneous rupture of membranes, or fetal movements. Her body mass index (BMI) was 31.3; rest of her vitals was within normal limits. The cervical ostium was dilated to 5 centimeters and was 100% effaced, station was +3; membranes were bulging, and the presenting part was high and not palpated. The prenatal labs were within normal limits. The patient was admitted to the labor and delivery unit and started to push. Antenatal steroid therapy was not administered due to the rapid progression to delivery. The patient delivered an extremely preterm viable female at 23 weeks with an intact placenta contained within the amniotic sac as a unit “en caul.” The amniotic sac was immediately torn, and a female newborn was extracted, emergently assessed, and managed by a neonatologist. The amniotic fluid was clear with no visible abnormalities. The delivery of the infant with placenta took 26 minutes. The newborn's Apgar scores were 4, 6, and 8 at 1, 5, and 10 minutes, respectively. No lacerations or tears were noted, and the fundus of the uterus was firm. The female newborn was transferred via airlift to a regional tertiary care hospital for emergent management of extreme prematurity, respiratory distress, thermoregulation, and the possibility of sepsis. The female infant began to deteriorate at day 4 after birth. She was intubated, ventilated, placed on an insulin drip for hyperglycemia, and given a stress dose of hydrocortisone and dopamine for hypotension. Broad-spectrum antibiotics were administered for probable sepsis, and blood cultures were drawn. Nitric oxide was administered at this point due to pulmonary hypertension with maximal setting and 100% fraction of inspired oxygen (FiO2); the infant had an oxygen saturation of 63%. Thereafter, she was transfused with packed red blood cell (PRBC) and platelets due to anemia and thrombocytopenia. An urgent head ultrasound displayed a severe grade three intraventricular hemorrhage bilaterally with an early grade four bleed in the left cerebral hemisphere. With a small window for survival and likely devastating neurological deficit, care was withdrawn from the infant after agreement between the parents and physicians. The child was pronounced dead at day 5 of life.

## 3. Discussion

Although en caul births are exceedingly rare, we provide here clinical guidelines covering the scope of emergent management and treatment of possible complications of en caul birth. It is well known that the caul itself is generally harmless and easily removed.

However, if a situation arises where rapid rupturing of membranes is required, especially using sharp forceps or scissors, we advise caution. Sharp instrumentation can pierce or damage the delicate neonatal skin, which may result in permanent scarring. Premature neonates have particularly labile skin, so clinical dexterity during removal process is advised. The majority of en caul deliveries occur in premature neonates [2–4]. En caul delivery may serve a clinically protective function in this high-risk cohort. In a comparative analysis with premature vaginal non-en caul deliveries, the benefits of en caul delivery are manifold. First, intact amniotic membranes provide a protective buffer from mechanical forces, such as potential bruising injuries and other examples of labor and delivery trauma during strong uterine contractions. Cesarean delivery en caul is an effective and easy technique for extremely preterm fetuses to protect them from pressure trauma and also results in less uterine injury [1]. Other benefits include the opportunity to complete a course of steroids, high cord pH, higher 5-minute Apgar scores for extremely preterm infants, protection from cord prolapse, and decreased risk of entrapment of the head in the setting of an insufficiently dilated cervix [4–6].

We conducted a review of the en caul case literature from 1975 to 2018, via PubMed, index words “en caul,” “enclosed in a amniotic sac,” “born in a caul,” English only, and excluding meeting abstracts.

One case documents vaginal en caul birth at 17 weeks of a nonviable fetus to a 35-year-old with no postpartum complications; unfortunately, the case had no further relevant details [2]. Another report describes a birth at 22 weeks and 6 days to a nulliparous woman; the infant died at 23 hours due to intraventricular hemorrhage and sepsis [5]. A third case outlines a birth at 23 weeks and 2 days to a 1 + 2 para woman; the infant died at 19 hours due to pulmonary hemorrhage [5]. There is also mention of a birth at 23 weeks and 5 days to a 0 + 2 para woman; the infant was discharged at 104 days of life with no further information available regarding her health [5]. One short case report from 1975 describes en caul birth at 34 weeks, living in the caul for 25 minutes on its way to the hospital and then undergoing immediate resuscitation. Three years after the case, when the article was published, no complications were identified that could be connected to being en caul [7]. A final case recounts a 23-week infant born via Cesarean section who lived without complications [8].

Respiratory distress, sepsis, and hemorrhagic complications are common postpartum sequelae after en caul birth. From these cases, however, it can be drawn that the preterm complications of en caul are similar to those of preterm non-en caul. The link between en caul birth and neonatal survival time is poorly understood at the current moment. Besides prematurity, these published cases do not mention other risk factors. Gravidity has not been previously proposed as a risk factor for en caul birth with postpartum complications. To the best of our knowledge, our case of en caul birth has the highest gravida (12) that has been reported; most other en caul cases have low gravida. We conclude that en caul birth may be an expected physiologic presentation and a rare variance that occurs randomly without particular effect.

This was a valuable teaching case for our institution. We advise obstetricians, neonatologists, and labor and delivery staff to conduct an immediate head ultrasound postpartum in extremely preterm en caul birth cases especially less than 24 weeks to rule out possibly fatal cerebral hemorrhagic complications. It may be possibly beneficial to leave the neonate in the caul due to the higher cord pH associated with en caul [1, 6]. However, guidelines have not been established on the duration of time a neonate may be en caul; this should be determined on a neonate-by-neonate case basis. Neonatologist consultation, respiratory intervention, aggressive preterm management, and neonatal intensive care unit (NICU) services should be considered.

## Data Availability

Any data will be made available on request.

## Abbreviations

TOP: Termination of pregnancies
NSVD: Normal spontaneous vaginal deliveries
GERD: Gastroesophageal reflux disorder
FiO2: Fraction of inspired oxygen
PRBC: Packed red blood cell
NICU: Neonatal intensive care unit.

## References

[1] Lin C.-H., Lin S.-Y., Yang Y.-H. (2010). Extremely Preterm Cesarean Delivery "En Caul".

[2] Greenstein J., Panzo W., O'Connor J., Hahn B. (2016). En Caul delivery.

[3] Abouzeid H., Thornton J. G. (1999). Pre-term delivery by Caesarean section 'en caul': A case series.

[4] Jin Z., Wang X., Xu Q., Wang P., Ai W. (2013). Cesarean section en caul and asphyxia in preterm infants.

[5] Richmond J. R., Morin L., Benjamin A. (2002). Extremely Preterm Vaginal Breech Delivery en Caul.

[6] Stoelinga B., Bienstock J., Goodwin C., Hueppchen N. (2006). En caul delivery of extremely preterm infants: Does it make a difference?.

[7] Heggarty H., Shenouda D., Grisdale M. (1975). Born in a Caul: Remarkable Survival.

[8] Prabakar C., Nimaroff M. L. (2012). Perfectly packaged: Upon delivery, the infant was still enclosed in the amniotic sac.

